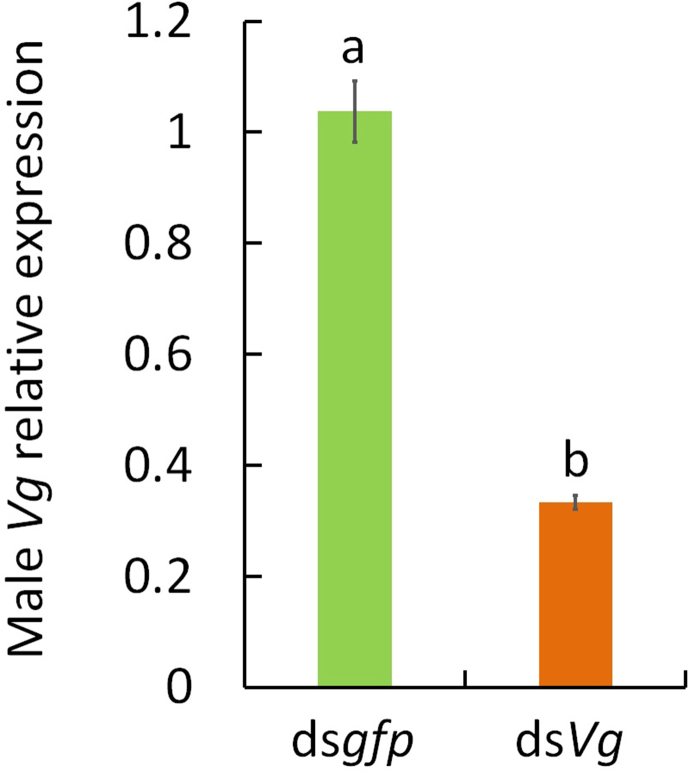# Testosterone maintains male longevity and female reproduction in *Chrysopa pallens*

**DOI:** 10.1016/j.heliyon.2024.e32478

**Published:** 2024-06-05

**Authors:** Xiaoping Liu, Xingkai Guo, Tingting Zhang, Jiaqi Duan, Lisheng Zhang, Mengqing Wang, Yuyan Li, Zhongjian Shen, Jianjun Mao

**Affiliations:** a Key Laboratory of Natural Enemy Insects, Ministry of Agriculture and Rural Affairs, Institute of Plant Protection, Chinese Academy of Agricultural Sciences, Beijing, PR China; b School of Advanced Manufacturing, Fuzhou University, Jinjiang, 362251, PR China

## Abstract

Vertebrate testosterone, an androgen present in the testes, is essential for male fertility. Vertebrate-type steroid hormones have been identified in insects, but their function remains unknown. Insect vitellogenin (Vg) is usually a female-specific protein involved in reproductive processes. However, males of some species, such as the green lacewing *Chrysopa pallens*, have Vg. Here, we demonstrated that the knockdown of *C. pallens* male *Vg* by RNAi significantly shortened the lifespan of males, suppressed the reproduction of post-mating females, and strikingly reduced the abundance of several immune-related compounds, including testosterone. LC-MS/MS revealed that *C. pallens* male testosterone had the same structure and molecular mass as vertebrate testosterone. Topical testosterone application partially restored the lifespan of *Vg*-deficient males and the reproduction of post-mating females. These results suggest that vertebrate-type testosterone maintains male longevity and female reproduction under the control of the male *Vg* in *C*. *pallens*.

## 1 Introduction

Insect spermatogenesis is regulated by hormones, such as 20-fydroxyecdysone (20E) and juvenile hormone (JH) [1,2]. It has been demonstrated that high levels of 20E promote the mitotic division of spermatogonia. However, JH generally exerts an inhibitory effect on spermatogenesis. JH also controls the development and secretion of male accessory glands. The levels of certain secretory peptides in the glands are increased by JH and reduced by 20E [2,3]. Recently, more than a dozen vertebrate-type steroids were identified in insects using specific and sensitive analytical techniques such as gas chromatography-mass spectrometry and radioimmunoassay [4,5]. For example, eleven non-ecdysteroid steroids such as testosterone, 5α-dihydrotestosterone, 11β-hydroxytestosterone and 11-ketotestosterone were identified in the hemolymph of *Sarcophaga bullata* (Diptera: Sarcophagidae) larvae [6]. Both C21 and C19 steroids were identified and quantified in the hemolymph of *Leptinotarsa decemlineata* [7]. Two typical fragments of testosterone were identified at the proper retention time and with the right abundance ratios when the contents of testosterone, pregnenolone, and estradiol in ovaria and testes of the locust *Locusta migratoria* were measured using gas chromatography combined with mass spectrometry [4]. Taken together, the presence of these steroids in insects is remarkable as it implies that the ‘vertebrate-type’ steroid hormone system is very ancient and may be shared by all phyla of animal kingdom. However, up to now, no correlations are proved between steroid titer fluctuations and divergent physiological processes. The vertebral-like steroids present in insect tissue extracts have not been shown to have a physiological role. The primary reason is that application of the typical steroids of higher vertebrates did not cause significant effects on insect physiology [8,9].

Most vertebrate testosterone is produced in the testes from the substrates, cholesterol and acetate. At least four steroidogenic enzymes are required for testosterone synthesis in Leydig cells: cholesterol side chain cleavage enzyme (CYP11A1) [10], HSD3B [11], CYP17A1 [12], and HSD17B3 [13]. However, the major enzymatic steps relevant to synthesis and catabolism, as revealed by studies with tissues of vertebrates, are not, or rarely observed in insects. The current conclusion is that typical vertebrate-type steroids presumably do not serve as physiological active substances in insects [9]. In *L. migratoria*, metabolic enzymes such as 17β-hydroxy steroid dehydrogenase (HSD), 20 α-HSD and 20 β-HSD are found. However, important biosynthetic enzymes such as 17α-hydroxylase and aromatase are absent, demonstrating that the biosynthesis of vertebrate-type steroids in insects does not follow the commonly used pathways in vertebrates [14].

Previously, the metabolism of vertebrate-type steroids was usually analyzed in a straightforward manner. The steroids were directly administrated to insects or insect tissues and then the metabolites were identified. In most of these studies, radioactive labelled steroids were used as precursors for a high sensitivity. Therefore, conversions of a tiny amount of steroids can easily be detected. These amounts may exactly match the physiological concentrations of endogenous substrates. In most *in vivo* studies, vertebrate-type steroids were delivered to insects by injection or feeding [15,16]. When progesterone was injected into *S*. *bullata* larvae, its metabolism into 17α-hydroxy-progesterone and testosterone was detected by chromatographic methods [15]. However, detection of the steroid metabolites in these insects is not necessarily an indication of their physiological relevance.

In most insects, vitellogenin (Vg) synthesis and deposition in developing oocytes during oogenesis are crucial for successful female reproduction [17,18]. These processes are also modulated by endocrine hormones such as JH, ecdysteroids, and neuropeptides [[19], [20], [21], [22]]. The roles of JH and ecdysteroids in controlling reproduction vary among insect groups. JH is the major hormone that regulates vitellogenesis in hemimetabolous insect orders, including Orthoptera, Blattodea, and Hemiptera. However, reproduction in the insect orders Diptera, Hymenoptera, and Lepidoptera is governed by ecdysteroids [[23], [24], [25], [26]].

Vg is a fundamental protein that is indispensable for reproduction. Its primary function is to supply nutrients and functional substances such as amino acids, fats, carbohydrates, phosphorus, and sulfur to developing oocytes [27]. It has long been established that insect Vg is usually female-specific. However, a male *Vg* gene was recently identified in the green lacewing, *Chrysopa pallens* (Rambur) (Neuroptera: Chrysopidae). The *C. pallens* male Vg has conserved cysteine residues, GL/ICG, RXXR, and other specific domains, similar to Vgs in other insects. Its expression level was stable at different ages but was significantly higher in the abdomen than in the head and chest [28]. *C*. *pallens* is one of the most prolific predators of insect pests, including various aphids, caterpillars, and larvae and eggs of other insects in the world's most agriculturally important regions [29,30]. These insect pests damage leaves, flowers, and fruits of many economically important crops, such as rice, maize, wheat, cotton, soybean and vegetables, and transmit a variety of destructive plant viruses. *C*. *pallens* has been extensively studied for its biological control potential for development of integrated pest management strategies to reduce the use of chemical insecticides in agricultural areas of the world [[31], [32], [33], [34]].

In this study, we found that *C. pallens* male *Vg* regulated male life expectancy and post-mating reproduction by maintaining the synthesis of immune-related compounds, including vertebrate-type testosterone. Topical testosterone application significantly recovered the life expectancy of *Vg*-deficient males and reproduction of post-mating females, suggesting that vertebrate-type steroid maintains male longevity and female reproduction under the control of male vitellogenin in *C. pallens*.

## 2 Material and methods

### 2.1 Insects

*C. pallens* culture used in the study was collected from the Beijing suburbs (40 that vertebrate-typeand reared in a greenhouse for more than 20 generations after identification using an EZ4 dissecting microscope (Leica, Wetzlar, Germany). *C. pallens* was fed pea aphids, *Acyrthosiphon pisum* (Hemiptera: Aphididae) reared on broad beans, *Vicia faba* L. (Fabales: Leguminosae) and kept at 25 °C with a photoperiod of 16:8 h light/dark cycle and a relative humidity of 70 %.

### 2.2 RNA interference (RNAi)

Male *C. pallens Vg* was depleted using dsRNA-mediated RNAi, as previously described [30,35,36]. A unique target fragment of 469 bp showing very low homology with other insect *Vg*s was selected from the C-terminus of the male *C. pallens Vg* transcript. Total RNA was isolated from the whole body of *C. pallens* adult male using TRIzol™ reagent (ThermoFisher, Waltham, MA, USA) on day six after eclosion. First-strand cDNA was synthesized using TransCript First-Strand cDNA Synthesis SuperMix (Transgene, Beijing, China). T7 primers (T7 promoter plus exon-specific sequence) were designed to amplify the *Vg* and *gfp* fragments. In vitro dsRNA synthesis was performed using the MEGAscript® RNAi Kit (Ambion, Austin, TX, USA). Template DNA and ssRNA in the dsRNA solution were removed by DNase/RNase digestion. Primers used are listed in Supplementary Table S1.

*C. pallens* cocoons of wild type were maintained under standard rearing conditions. Once adults emerged, males and females were distinguished by observing the externalia and kept separated. The dsRNA (2 μg, 2 mg/ml) was injected into 2-day-old male adults using an Eppendorf InjectMan NI 2 and FemtoJet express microinjector system (Eppendorf, Hamburg, Germany). Each male was injected at the joint of the fourth and fifth abdominal segments and housed with a virgin female. Thirty-four to thirty-seven male adults were injected for each dsRNA. The male survival rates were recorded daily until all males died. When female adults began to oviposit, the eggs were counted, and lifetime fecundity was calculated.

### 2.3 Quantitative PCR (qRT-PCR)

Gene transcript levels were examined by qRT-PCR. Using a total reaction volume of 25 μL, the mixture contained 200 nM each of forward and reverse gene-specific primers, 11.25 μL Go Taq qPCR Master Mix (Promega, Madison, WI, USA), and cDNA produced from 2 μg total RNA. qRT-PCR was conducted on a LightCycler® 96 (Roche, Basel, Switzerland) using the following thermal program: initial incubation at 95 °C for 60 s, followed by 40 cycles of 95 °C for 15 s and 60 °C for 30 s. The housekeeping gene actin from *C. pallens* was used as an internal control for normalization of gene expression. Four technical replicates were used for each treatment. Relative mRNA levels were quantified using the 2^−ΔΔCt^ approach [37]. The specific primers for the tested genes are listed in Table S1.

### 2.4 Microscopy of female ovaries

On day six post-emergence, three females were randomly selected for each dsRNA treatment. The ovaries were dissected and observed at 20-fold magnification using a VHX-2000 digital microscope (KEYENCE, Neu-Isenburg, Germany) as previously described [30]. Three ovarioles were randomly selected from a single ovary and the length of primary follicles was measured.

### 2.5 Quasi-targeted metabolomics analysis

On day 5 post-eclosion, the dsRNA-treated males were dissected in PBS, and the terminal 3.5 abdominal segments containing the entire reproductive tract were cut from the body and ground in liquid nitrogen. The homogenate (100 mg) was suspended in pre-chilled 80 % methanol (500 μL) with vortexing and centrifuged at 15,000×*g* for 20 min at 4 °C. The supernatant was diluted with methanol to a final concentration of 53 %. The LC-MS/MS analysis was performed using an Exion LC system (SCIEX, Framingham, MA, USA) coupled to a QTRAP 6500 Plus spectrometer (SCIEX). An Xselect HSS T3 column (Waters, Framingham, MA, USA) was used to separate the extracts with a solvent gradient at a flow rate of 0.4 ml/min. The solvent gradient was as follows: 2 min, 98 % A; 15 min, 98-0% A; 17 min, 0 % A; 17.1 min, 0–98 % A; 20 min, 98 % A. The eluents used for the positive/negative polarity mode were eluent A (0.1 % formic acid in water) and eluent B (0.1 % formic acid in acetonitrile). Principal component analysis (PCA) and partial least squares discriminant analysis (PLS-DA) were performed using the MetaX software (http://metax.genomics.cn/). The KEGG, HMDB, and LIPIDMaps databases were used to annotate the identified metabolites. Five to six replicates were analyzed for each dsRNA treatment.

### 2.6 Testosterone measurement

Testosterone samples were prepared using liquid-liquid extraction (LLE) [38]. The abdomens of ten male *C. pallens* were ground in liquid nitrogen on days 0, 10, and 15 post eclosion. The homogenate (100 mg) was suspended in 200 μl buffer A (0.5 mol/L ammonium acetate; pH, 5.5) and mixed at room temperature for 5 min. The LLE was performed twice using a mixture of ethyl acetate and n-hexane solution (3:2, v/v). The upper organic phase was transferred and evaporated to dryness using nitrogen blowing apparatus. The extract was dissolved in 200 μl of basic buffer (0.2 mol/L sodium carbonate; pH, 9.8). The solution was extracted twice using n-hexane (500 μl each time). The organic phase was dried at 40 °C and redissolved in 100 μl of methanol for mass spectrum analysis.

To confirm the presence of testosterone in *C. pallens* males, LC-MS/MS was performed by a Vanquish™ Flex UHPLC system with a Q Exactive™ GC Orbitrap™ mass spectrometer (Thermo Fisher Scientific™, Waltham, MA, USA) as previously described [39]. Testosterone (≥98.33 %) was purchased from Dr. Ehrenstorfer (Augsburg, Germany) and used as the calibration standard. Testosterone was separated on an Accucore™ Vanquish™ C18 + UHPLC column (1.5 μm, 100 × 2.1 mm, Thermo Fisher Scientific™) kept at 30 °C using a gradient program. Solvents A and B comprised 0.1 % formic acid in water and acetonitrile, respectively, with program 0–20 min, 5%–95 % B; 20–22 min, 100 % B, 22–25 min, 5 % B. The flow rate was maintained at 0.4 ml/min and the injection volume was 5 μl.

To quantify testosterone in *C. pallens* males at different developmental stages, UPLC-MS analysis was performed with an ACQUITY UPLC BEH C18 column (2.1 × 100 mm, 1.7 μm) (Waters) using a gradient program. Three replicates were analyzed for each stage. Solvents A and B comprised 0.1 % formic acid in water and acetonitrile, respectively with a program of 0–5 min, 40%–65 % B; 5.25–7.25 min, 100 % B, 7.5–10 min, 40 % B. The flow rate was maintained at 0.3 ml/min. The injection volume was 5 μl and the samples were kept at 4 °C in the autosampler.

### 2.7 Testosterone treatment

To test the recovery of fecundity by testosterone, a testosterone reagent (Virtue-Clara, Beijing, China) was solubilized in acetone at 20 mg/ml. Two micrograms of ds*Vg* were injected into males on day 2 post-emergence and then testosterone solution (1 μl) was dropped onto the joint of the fourth and fifth abdominal segments of males on days 3 and 4 post-emergence for easy absorption. Two control groups were established in this study. One comprised males treated with 2 μg of ds*Vg* on day 2 post-emergence and treated with 1 μl of acetone on days 3 and 4 post-emergence. The other consisted of males treated with 2 μg of ds*gfp* on day 2 post-emergence and treated with 1 μl of acetone on days 3 and 4 post-emergence. After the second testosterone and acetone treatments, each male was kept with a virgin female (2-day-old) and reproductive parameters were recorded.

## 3 Results

### 3.1 Male Vg is crucial for male lifespan and female reproduction

**Fig. 1:**
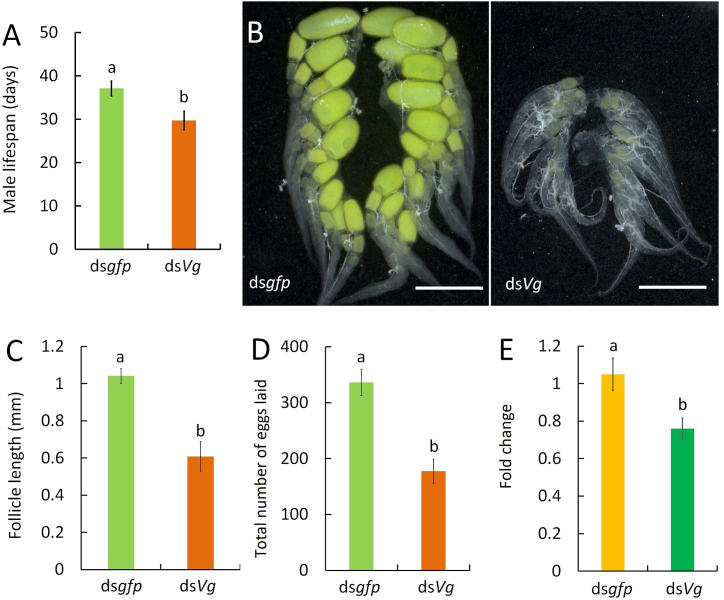
Knockdown of male *Vg* shorted male lifespan and impaired female reproduction. Male life expectancy was significantly shortened by *Vg* knockdown (**A**) (*t*-test, n = 34–37). Mating with ds*Vg*-treated males suppressed ovarian development (**B**), reduced primary follicle size (**C**) (*t*-test, n = 9) and reproductive output (**D**) (*t*-test, n = 34–37), and significantly reduced female *Vg* expression (**E**) (*t*-test, n = 4). B, scale bar = 1 mm. Bars (mean ± SE) with different letters indicate significant differences.

Male *Vg* transcripts were significantly reduced following ds*Vg* injection (Fig. S1) (*P* < 0.01). The lifespan of ds*Vg*-treated males was significantly shorter than that of ds*gfp*-treated males (Fig. 1A) (*P* < 0.01). Six days after emergence, females mated with ds*gfp*-treated males exhibited mature ovaries with vitellogenic primary follicles. However, ovarian development in females mated with ds*Vg*-treated males was arrested (Fig. 1B). The primary follicles in females mated with ds*Vg*-treated males were significantly smaller than those in females mated with ds*gfp*-treated males (Fig. 1C) (*P* < 0.01). Total fecundity (*P* < 0.01) (Fig. 1D) and *Vg* expression (Fig. 1E) (*P* = 0.02) in females were significantly impaired after mating with ds*Vg*-treated males.

### 3.2 Differentially accumulated metabolites after male Vg knockdown

**Fig. 2:**
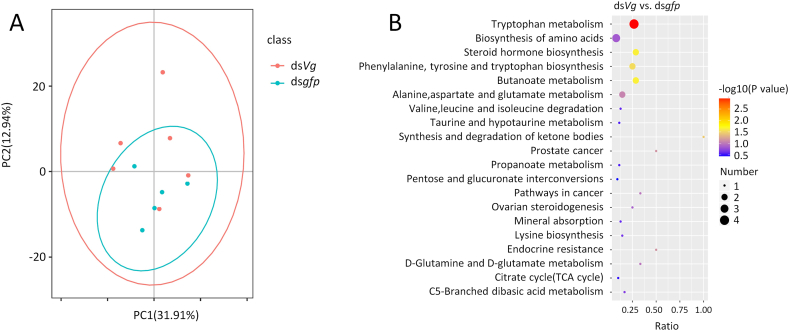
PCA plot and KEGG enrichment of differentially accumulated metabolites post male *Vg* knockdown. PCA plot of metabolite features in males treated with ds*Vg* and ds*gfp* (**A**). KEGG enrichment map of differentially accumulated metabolites post male *Vg* knockdown (**B**). In B, the vertical axis represents pathway entries and the horizontal axis represents the ratio of the number of differentially accumulated metabolites in the pathway to the total number of metabolites in the pathway. Circle size corresponds to the number of enriched metabolites.

Metabolome analysis yielded 425 and 361 compounds in positive and negative ion modes, respectively. The thresholds for significant differences in metabolite abundance were VIP >1, FC > 1.2, and *P* < 0.05. The principal component analysis (PCA) revealed that ds*Vg* and ds*gfp* had different effects on metabolite accumulation in the reproductive tracts (Fig. 2A). Kyoto encyclopedia of genes and genomes (KEGG) enrichment analysis of differential metabolites showed that five pathways, including tryptophan metabolism and steroid hormone synthesis, were enriched in the ds*Vg*-versus-ds*gfp* differential metabolites (Fig. 2B). The knockdown of male *Vg* led to the downregulation of 22 compounds. Among them, the level of the steroid hormone testosterone was reduced by 41.1 % following the knockdown of male *Vg*. The top ten downregulated metabolites are shown in Table 1.

**Table 1:** Differential metabolites post male *Vg* knockdown.

Compound ID	Annotation	Classification	Log_2_FC
Com_519_Pos	Dl-Threitol	Sugar Alcohols	−0.92843
Com_343_Neg	Phenylpyruvic Acid	Organic Acid And Its Derivatives	−0.86014
Com_155_Neg	Indoleacetaldehyde	Organoheterocyclic Compounds	−0.82646
Com_2_Neg	Indolelactate	Indole And Its Derivatives	−0.78699
Com_410_Pos	5-Hydroxytryptophol	Indole And Its Derivatives	−0.76389
Com_539_Pos	Testosterone	Hormones	−0.76328
Com_275_Neg	Alpha-Ketoglutaric Acid	Tca Cycle	−0.69826
Com_62_Neg	Acetoacetate	Organic Acid And Its Derivatives	−0.62387
Com_129_Neg	Alpha-Benzylsuccinic Acid	Organic Acid And Its Derivatives	−0.60988
Com_195_Neg	Thymidine 5′-Diphosphate	Nucleotide And Its Derivates	−0.60937

### 3.3 Identification of testosterone in adult males

**Fig. 3:**
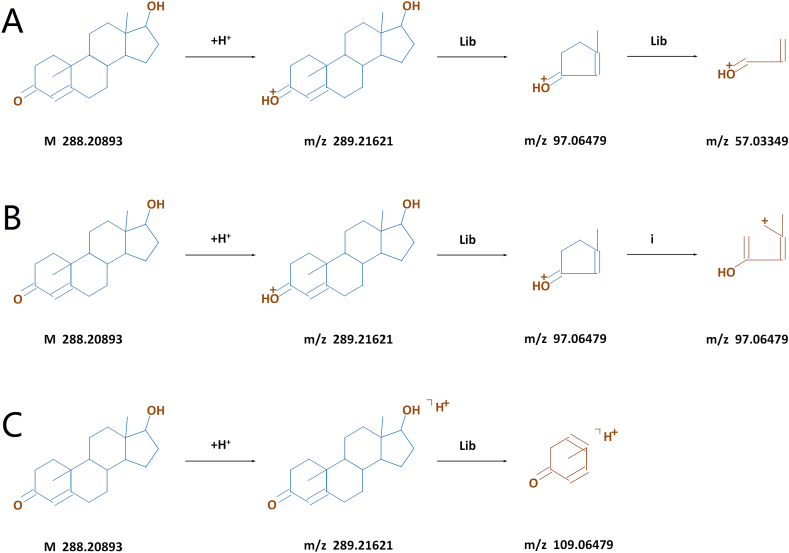
Structures of testosterone molecules and their cleavage into fragment ions revealed by LC-MS/MS. **A**, Charge of testosterone and its cleavage at *m*/*z* 57.03. **B**, Charge of testosterone and its cleavage at *m*/*z* 97.04. **C**, Charge of testosterone and its cleavage at *m*/*z* 109.06.

Liquid chromatography-tandem mass spectrometry (LC-MS/MS) revealed the cleavage of testosterone molecules and the mass-to-charge ratio (*m*/*z*) of the testosterone ions. The testosterone molecules were first positively charged to produce a parent ion (*m*/*z* = 289.21), and then fractured to generate several fragment ions (*m*/*z* = 97.06, 57.03, and 109.06) (Fig. 3A–C).

**Fig. 4:**
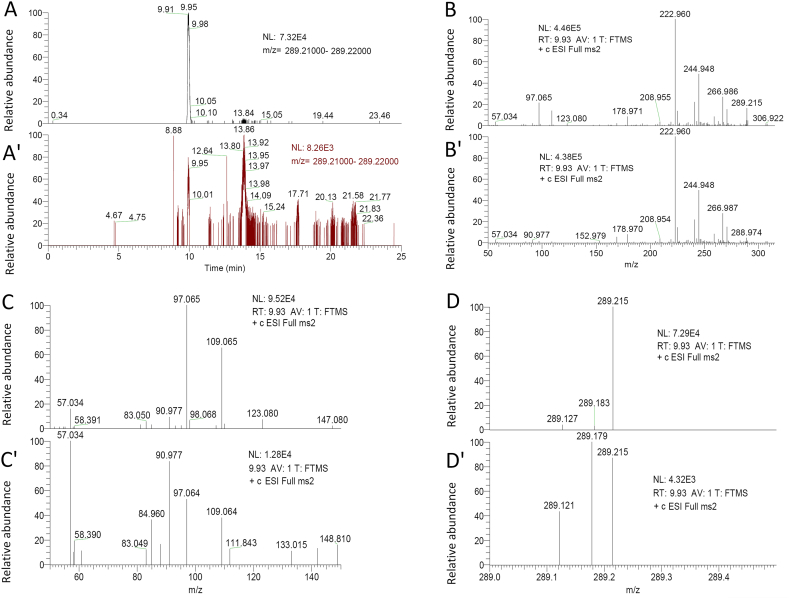
LC-MS/MS chromatograms of testosterone standard (5 ppm) and samples from 5-day-old *C. pallens* males. **A**, Extracted ion chromatogram (EIC) of testosterone standard. **A′**, EIC of testosterone sample. **B**, Secondary ion mass spectrum of testosterone standard. **B′**, Secondary ion mass spectrum of testosterone sample. In B and B′, the testosterone fragment ion at *m*/*z* 57.034 is shown. **C**, Secondary ion mass spectrum obtained from monitoring the testosterone standard transition for *m*/*z* 50–150. **C′**, Secondary ion mass spectrum obtained from monitoring the testosterone sample transition for *m*/*z* 50–150. In C and C′, three testosterone fragment ions were detected at *m*/*z* 57.03, 97.06 and 109.06. **D**, Secondary ion mass spectrum obtained from monitoring the testosterone standard transition for *m*/*z* 289.0–289.5. **D′**, Secondary ion mass spectrum obtained from monitoring the testosterone sample transition for *m*/*z* 289.0–289.5. In D and D′, the positively charged parent ion of testosterone is shown (*m*/*z* = 289.21).

Extracted ion chromatography (EIC) of the testosterone standard showed a testosterone retention time of 9.95 min (Fig. 4A). The EIC of the testosterone sample from 5-day-old males showed multiple chromatographic peaks due to the presence of impurities in the sample, including an obvious peak at 9.95 min (Fig. 4A’). The testosterone fragment ion at *m*/*z* 57.034 was detected in testosterone standard and sample (Fig. 4B and B′). Furthermore, both the testosterone standard and sample in 5-day-old males contained ions at *m*/*z* 57.03, 97.06, 109.06 (Fig. 4C and C′), and 289.21 (Fig. 4D and D’). These results suggest that testosterone is present in 5-day-old *C. pallens*.

**Fig. 5:**
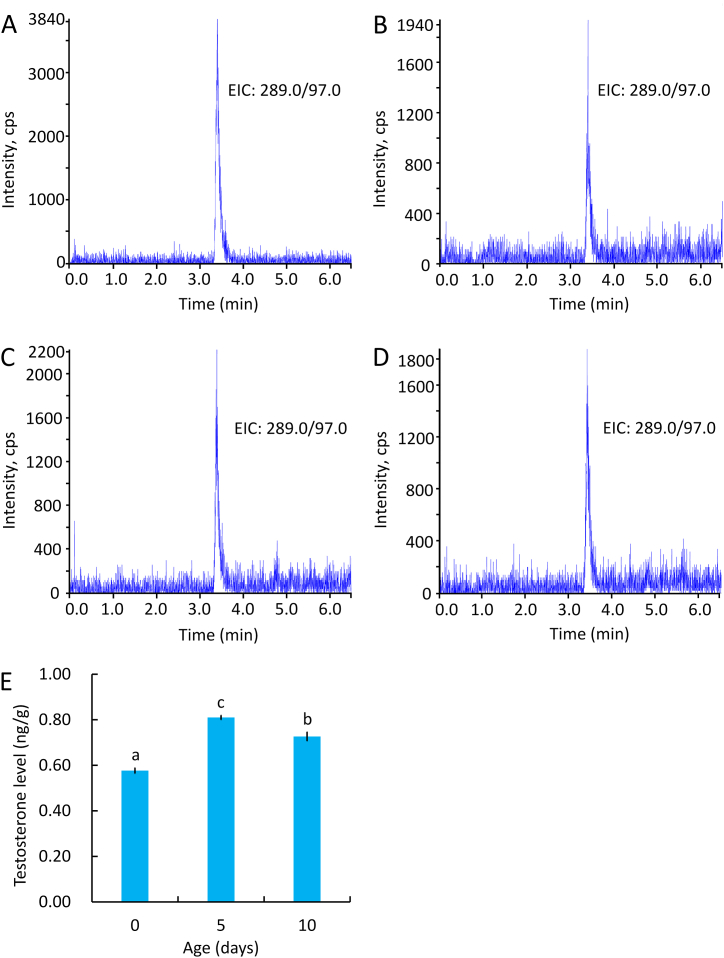
Quantification of testosterone in *C. pallens* males using UPLC-MS. **A**, EIC of testosterone (0.8 ng/ml) for calibration. **B**, EIC of testosterone in 0-day-old males. **C**, EIC of testosterone in 5-day-old males. **D**, EIC of testosterone in 10-day-old males. **E**, Testosterone level in *C. pallens* males (one-way ANOVA, n = 3).

*C. pallens* males at different developmental stages synthesized testosterone (Fig. 5A–D). The testosterone level on days 0, 5, and 10 post emergence were 0.58 ± 0.01, 0.81 ± 0.01 and 0.73 ± 0.02 ng/g) (Fig. 5E) (F = 65.45, *P* < 0.01).

### 3.4 Restoration of fecundity and longevity by testosterone

**Fig. 6:**
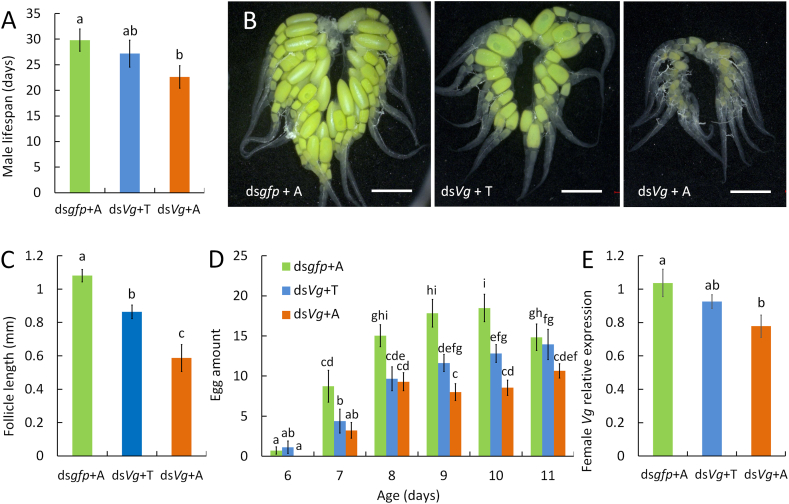
Restore of male longevity and female reproduction by testosterone. Female Reproduction is partially restored by topical testosterone application in males. Male lifespan (**A**) (one-way ANOVA, n = 29–32), female ovarian development (**B**), primary follicle size (**C**) (one-way ANOVA, n = 9), total fecundity (**D**) (Generalized Linear Model, n = 29–32) and *Vg* expression on day 10 post emergence (**E**) (one-way ANOVA, n = 4) post mating were recovered substantially. Scale bar = 1 mm. A, acetone; T, testosterone. Bars (mean ± SE) with different letters represent significant differences.

Topical application of testosterone to ds*Vg*-treated males restored their longevity. The male longevity in the ds*gfp*-acetone-treated group was significantly longer than that in ds*Vg*-acetone-treated group, but not significantly longer than that in ds*Vg*-testosterone-treated group (Fig. 6A) (F = 2.27, *P* = 0.11). The use of testosterone in ds*Vg*-treated males also rescued post-mating ovarian development (Fig. 6B) and significantly increased the primary follicle size (Fig. 6C) (F = 19.13, *P* < 0.01). Reagent, age, and their interaction had significant effects on the daily fecundity of females mated with ds*Vg*-treated males (reagent: df = 2, *P* < 0.01; age: df = 5, *P* < 0.01; age × reagent: df = 10, *P* = 0.01) (Fig. 6D). Relative expression of *Vg* in females mated with ds*Vg*-treated males was partially recovered by the use of testosterone in males (F = 5.28, *P* = 0.03) (Fig. 6E).

## 4 Discussion

In recent years, LC-MS/MS has been widely used for testosterone measurement. Despite its high specificity, mass spectrometry has drawbacks in terms of testosterone measurements. For example, because other endogenous compounds are similar in structure and molecular mass, testosterone must be chromatographically separated for accurate quantification. LC-MS/MS analysis of *C. pallens* testosterone was performed following the sensitive method previously described [39]. This method was established to detect total testosterone levels in all patients. The accuracy was verified using the American National Institute of Standards and Technology Standard Reference Material 971. In the present study, vertebrate-type testosterone in *C. pallens* males was identified using quasi-targeted metabolomic analysis. Furthermore, LC-MS/MS revealed that both the testosterone standard and sample in *C. pallens* males contained ions at *m*/*z* 57.03, 97.06, 109.06 (Fig. 4C–F), and 289.21 (Fig. 4G–H), demonstrating that vertebrate-type testosterone was present in *C. pallens* males. Now, the origin of vertebrate-type steroids in insect tissues still remains concerns. One hypothesis is that they are simply derived from dietary source of the insects [9]. This hypothesis is less likely to hold in the present study because the males treated with ds*Vg* and ds*gfp* were fed the same diet in the assay, and the vertebrate-type testosterone level in *C. pallens* males should be independent of dietary source.

The average concentration of total testosterone for all human age groups is 15.36 ± 4.86 nmol/L [40]. That is approximately 4.43 ± 1.40 ng/g. However, the abdominal vertebrate-type testosterone of 5-day-old *C. pallens* males was 0.81 ng/g, a concentration much lower than that in humans. Taken together, these results suggest that *C. pallens* can synthesize the vertebrate androgen testosterone, in addition to common hormones such as JH and 20E. Since the first isolation and synthesis of the testosterone molecule in 1935, vertebrate testosterone has been studied for nearly a century [41]. In the past decades, the endocrine system in insects is found much more intricate than previously deemed and sharing many common characteristics with that in vertebrates. The classical sex steroid hormones of higher vertebrates, including progesterone, testosterone, estradiol, has been identified in insect tissues [4,5,42,43]. So far, biosynthesis of vertebrate-type steroids from cholesterol has been documented in two insects, the water beetle *Acilius sulcatus* (Coleoptera: Dytiscidae) and the tobacco hornworm *Manduea sexta* (Lepidoptera: Sphingidae). The vertebrate-type steroids in the prothoracic glands of *A. sulcatus* are linked to defense against vertebrate predators [44,45]. The tobacco hornworm *M. sexta* has no distinct defensive glands. However, high amounts of a vertebrate-type steroid conjugate, i.e. 20β-diol glucoside, were isolated from ovaries and eggs. To date, nothing is known about a physiological function of the C21 steroid conjugate in ovaries and eggs of this species [46]. In the present study, the abundance of *C. pallens* vertebrate-type testosterone in males is closely associated with longevity of males and fecundity of post-mating females, suggesting its crucial roles in regulation of insect life-span and reproduction.

Women around the world live longer than men. One possible mechanism leading to sex differences in longevity in humans is that testosterone has a depressing effect on the immune system [40]. However, in the present study, treatment with testosterone significantly extended the life expectancy of *Vg*-deficient males, demonstrating that the *C. pallens* testosterone and its counterparts in humans have opposite effects on life expectancy. Topical treatment with testosterone positively affected *C. pallens* male life span and female reproduction. Although these two events occurred in different sexes, their trends followed the same direction. In most animals, longevity is achieved at the expense of fertility, but social insects, such as the ant *Cardiocondyla obscurio* (Hymenoptera: Formicidae) and queen honeybee *Apis mellifera* (Hymenoptera: Apidae), do not have this tradeoff [47,48]. Therefore, life expectancy is positively correlated with the reproductive performance of social insects. This phenomenon was also observed in *C. pallens*.

Insect Vg is generally known as a female-specific protein fundamental to female reproduction. Males of a small number of species, such as honeybees, have Vg, and its function is associated with social organization [49]. Here, knockdown of *C. pallens* male *Vg* significantly shortened the male lifespan and impaired female reproduction, suggesting novel roles for insect Vg. In queen bees, Vg acts as an antioxidant that extends longevity [50]. We conclude that the *C. pallens* male Vg regulates lifespan via a similar mechanism.

Metabolome profiles indicated that in addition to testosterone, dl-threitol, phenylpyruvic acid, indoleacetaldehyde, indolelactate, and 5-hydroxytryptophol were also downregulated by treatment with ds*Vg* (Table 1). To date, the threitol biosynthetic pathway has not been fully characterized in most organisms. In the Alaskan beetle *Upis ceramboides* (Coleoptera: Tenebrionidae), threitol accumulation is associated with cold tolerance [51]. In the present study, dl-threitol may be linked to tolerance of adverse conditions in *C. pallens* males. Phenylpyruvic acid is an intermediate or catabolic by-product of phenylalanine metabolism [52]. In malarial mosquitoes, phenylalanine metabolism regulates reproduction and immunity. Silencing of phenylalanine hydroxylase (PAH), which is involved in the conversion of phenylalanine to tyrosine, significantly reduces the number of eggs, retards vitellogenesis, and impairs melanization of the chorion [53]. However, the role of the phenylalanine metabolism in male insects remains unclear. Here, we demonstrated that it is also associated with vitellogenesis and immunity in *C. pallens* males.

KEGG analysis revealed that tryptophan metabolism was enriched in metabolites that differentially accumulated between ds*gfp*-treated and ds*Vg*-treated males (Fig. 2B). Indoleacetaldehyde and indolelactate are distributed in this pathway. In the human body, tryptophan and its metabolites affect immune reactions, possess antioxidant properties, and act as anabolic signals [54]. In insects, tryptophan is the initial precursor for the formation of ommochromes, which regulate the color of insect wings and eyes [55,56]. In the present study, it was difficult to explain the downregulation of tryptophan metabolism after disruption of male *Vg*. We speculate that *C. pallens* male *Vg* is responsible for maintaining stable tryptophan metabolism that may be essential for immunity and spermatogenesis.

Briefly, among the metabolites differentially accumulated between in ds*Vg*-treated- and ds*gfp*-treated males, dl-threitol, phenylpyruvic acid, indoleacetaldehyde, indolelactate, and testosterone have been implicated in insect or vertebrate adversity tolerance and immunity. Their downregulation leads to a significantly shorter lifespan in Vg-deficient males.

## Data availability statement

No data was used for the research described in the article.

## Limitations of the study

A major limitation of our study is that we did not rule out the possibility that the vertebrate-type testosterone in male *C. pallens* may be derived from the dietary source. In this study, the testosterone level is about 41.1 % lower in *Vg*-deficient males than in ds*gfp* treated males. This possibility is less likely to hold because the *Vg*-deficient males and ds*gfp* treated males were fed the same diet, pea aphids. However, to completely rule out the possibility, the presence of vertebrate-type testosterone in pea aphids should be analyzed using LC-MS/MS. We can be sure that male *C. pallens* synthesize the vertebrate-type testosterone if the testosterone is not detected in pea aphids.

## CRediT authorship contribution statement

**Xiaoping Liu:** Methodology, Investigation, Formal analysis. **Xingkai Guo:** Methodology, Investigation. **Tingting Zhang:** Validation, Supervision, Formal analysis. **Jiaqi Duan:** Methodology, Investigation. **Lisheng Zhang:** Supervision, Project administration, Conceptualization. **Mengqing Wang:** Visualization, Supervision. **Yuyan Li:** Validation, Supervision. **Zhongjian Shen:** Visualization, Supervision. **Jianjun Mao:** Writing – review & editing, Writing – original draft, Supervision, Project administration, Funding acquisition, Conceptualization.

## Declaration of competing interest

The authors declare that they have no known competing financial interests or personal relationships that could have appeared to influence the work reported in this paper.

## Figures and Tables

**figs1:**