# Supplementary material for: Testosterone maintains male longevity and female reproduction in Chrysopa pallens
Source: Heliyon. 2024 Jun 5;10(11):e32478. doi: 10.1016/j.heliyon.2024.e32478 (PMC11201114; doi:10.1016/j.heliyon.2024.e32478)
Supplement: Multimedia component 1 [file mmc1.docx]

| **Table S1 Primers used in the paper.** | | | | |
| --- | --- | --- | --- | --- |
| Gene | PCR type | Forward | Reverse | Fragment size (bp) |
| Male Vg | RT-PCR^a^ | TAATACGACTCACTATAGGGGATATGGGTCATCGTCATG | TAATACGACTCACTATAGGGCTTCCTCCGAATCTTGCT | 434 |
| Male And Female *Vg* | RT-PCR^b^ | TCCATACTTAATTTGGGCTAC | CTTCCTCCGAATCTTGC | 123 |
| *gfp* | RT-PCR^a^ | TAATACGACTCACTATAGGGCACAAGTTCAGCGTGTCCG | TAATACGACTCACTATAGGGAGTTCACCTTGATGCCGTTC | 461 |
| *Actin* | RT-PCR^b^ | AACTTCCCGACGGTCAAGTCAT | TGTTGGCGTACAAGTCCTTACG | 196 |

^a^ Primers used in dsRNA synthesis for amplification of the target fragments.

^b^ Primers used in qRT-PCR for mRNA level measurement of different genes.
